# Two Live Births following Robotic-Assisted Abdominal Cerclage in Nonpregnant Women

**DOI:** 10.1155/2013/256972

**Published:** 2013-09-12

**Authors:** Ahmet Göçmen, Fatih Şanlıkan

**Affiliations:** Department of Obstetrics and Gynecology, Ümraniye Education and Research Hospital, Ümraniye, 34766 İstanbul, Turkey

## Abstract

*Introduction*. To report the robotic-assisted abdominal cerclage performed in two nonpregnant women and the success of live birth outcomes. *Presentation of Cases*. A 36-year-old woman with a complaint of recurrent second trimester pregnancy losses and a 35-year-old patient with a complaint of preterm deliveries and cervical insufficiency underwent robotic assisted abdominal cervicoisthmic cerclage placement in nonpregnant period. The two patients had spontaneous pregnancy after the robotic-assisted abdominal cerclage and delivered healthy infants. *Discussion*. The limitations of traditional laparoscopic abdominal cerclage have been accomplished with robotic surgery advantages especially intuitive movements and increased range of motion. There are only a few studies in the literature including robotic assisted abdominal cerclage in nonpregnant women, and only five successful live birth outcomes were reported. In this paper, we reported the sixth and seventh cases of achieved live pregnancy after robotic assisted abdominal cerclage in the literature. *Conclusion*. Robotic assisted abdominal cerclage is a good alternative surgical method with successful pregnancy outcomes.

## 1. Introduction

Cervical insufficiency is a condition which is characterized with painless cervical dilatation and is an important factor causing second trimester pregnancy loss. Cervical insufficiency occurs in 0.5–1% of all pregnancies [1]. Extensive cervical conization, routine dilation and curettage for diagnostic and therapeutic purposes, traumatic cervical lacerations during deliveries, and congenital or DES exposure-related abnormalities may lead to cervical insufficiency [2]. Cervical cerclage is a surgical procedure which involves suturing the neck of the cervix with a purse type stitch to keep the cervix closed for the treatment of cervical incompetence. The vaginal route is the most used surgical method for cervical cerclage. The abdominal approach is usually only done if the cervix is too short to attempt a standard cerclage, or if a vaginal cerclage has failed or is not possible. The minimal invasive techniques, such as laparoscopy, have been considered for the abdominal cerclage in the last few years [3–5]. Due to technical difficulties and limitations of the laparoscopic surgery, laparoscopic abdominal cerclage has not gained widespread popularity. Robotic surgery gives the opportunity to surgeons in order to perform operations requiring advanced suturing techniques and helps to eliminate the technical limitations of the laparoscopic surgery [6]. There are only a few case report studies including robotic assisted abdominal cerclage in the literature [7–9]. In this report, we presented the outcomes of two robotic abdominal cerclage cases.

## 2. Case Presentations

### 2.1. Case  1

**Figure 1:**
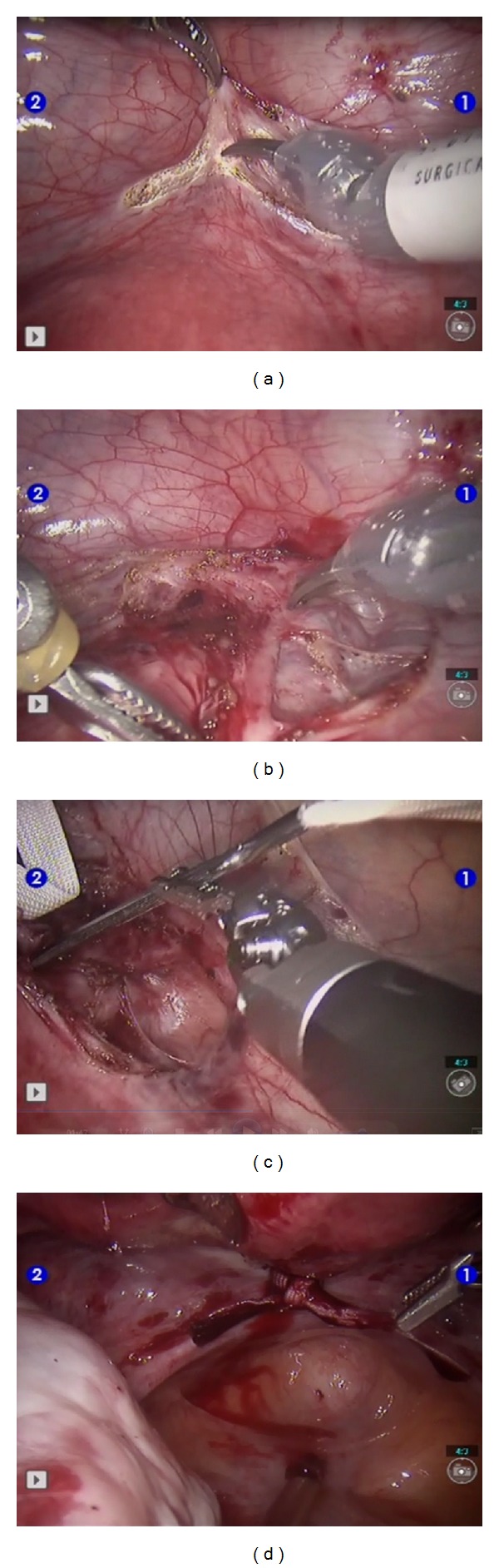
Robotic-assisted abdominal cerclage. (a) Preparation of the vesicouterine peritoneal flap, (b) dissection of the uterine artery, (c) passing of the suture, and (d) tying of the mersilene tape.

A 36-year-old woman with a history of gravida 4, para 0, was admitted to our clinic with a complaint of recurrent second trimester pregnancy losses (15th, 18th, 22nd, and 15th weeks of gestation). The patient had two unsuccessful vaginal cerclages during the last two pregnancies. The rest of her medical history was unremarkable. The physical examination revealed insufficient cervical tissue. We decided to perform robotic-assisted abdominal cerclage prepregnancy. The patient was appropriately counseled and a written informed consent was obtained. The robotic procedure was approved by the institutional review board at our hospital. The patient underwent a successful robotic assisted abdominal cerclage. Da Vinci S surgical system with four-trocar transperitoneal approach was used for the procedure. The surgical technique was as follows. The vesicouterine peritoneal flap was taken down, and it visualized the uterine vessels bilaterally. An avascular area between the uterus and the bifurcation of the uterine artery was prepared. A 5 mm mersilene tape with a flattened needle was sent to the abdominal cavity and was passed medially to the uterine artery from the anterior to the posterior direction. The same procedure was performed at the other side. The passed tape was tied firmly posteriorly. The surgical technique of robotic assisted abdominal cerclage is shown in Figure 1. The operation time and console time was 67 and 41 minutes, respectively. There was no intraoperative complication and the patient was discharged from hospital at the postoperative first day. During the followup of the patient, she had a spontaneous pregnancy about four months after surgery. The follow up of the pregnancy was done at another hospital in a different country, and she had a cesarean section and delivered a healthy infant.

### 2.2. Case  2

A 35-year-old patient with, gravida 2 presented to our clinic with a complaint of preterm deliveries. In her past medical history, she had two deliveries which were at the 20th and 26th weeks of gestation. On physical examination, the cervix was short in appearance, and the transvaginal 21 mm cervical length measurement supported the finding. The patient was offered to be operated with robotic surgery, for abdominal cerclage. After her acceptance, she underwent robotic surgery and the procedure was successfully completed in 43 minutes. The same surgical technique was used as in case  1. No surgical complications occurred during the intervention and the postoperative stay and no conversion to laparotomy. The patient was discharged from hospital one day after operation. The patient have a spontaneous pregnancy. The followup of the patient was uneventful, and she had a cesarean section at 38th week of gestation and delivered a healthy infant.

## 3. Discussion

Increased neonatal survival rates are associated with transabdominal cerclage [10]. However, the placement of a transabdominal cervical cerclage has been regarded as considerably more morbid than a transvaginal cerclage. Minimal invasive techniques such as laparoscopy and robotic assisted surgery in the treatment of an incompetent cervix are promising options [3, 7]. The limitations of traditional laparoscopic abdominal cerclage have been accomplished with robotic surgery advantages especially intuitive movements and increased range of motion. These properties allow surgeon to perform intracorporeal knot tying easily and perform tissue dissection more precisely than laparoscopy. 

There are only a few studies including robotic assisted abdominal cerclage in the literature. Barmat et al. reported a case which included the first robotic assisted abdominal cerclage in a nonpregnant woman [7]. The patient had a history of cervical insufficiency and was not a candidate for transvaginal cerclage placement due to extensive cervical conization history, the presence of short cervix, and second trimester loss of pregnancy after in vitro fertilization. Prepregnancy abdominal cerclage using the da Vinci robotic surgical system was performed. The mean operation time was 120 minutes with minimal blood loss. In this report, there was no information about the followup of the patient to know either further pregnancy status or successful live birth to assess the success of the robotic abdominal cerclage.

The first robotic-assisted laparoscopic cerclage in a pregnant patient was performed by Fechner et al. in 2009 [8]. The patient was at 12 weeks' gestation with a history of preterm delivery at 31 weeks' gestation and a cold-knife conization for cervical dysplasia which resulted in the removal of most of the vaginal portion of the cervix. An elective abdominal cerclage was performed with robotic-assisted laparoscopic technique. The remainder of the pregnancy was uncomplicated, and she delivered a healthy infant via elective cesarean section at 37 weeks' gestation.

The most comprehensive study including robotic assisted abdominal cerclage was reported by Moore et al. in which a total of 24 nonpregnant patients underwent robotic procedure [9]. Only in one case conversion to laparotomy due to dense adhesions was done. The mean operation and console times were reported to be 118 and 58 minutes, respectively. The mean length of hospital stay was less than one day. They compared the robotic surgical outcomes with laparoscopy and stated that although the robotic procedure takes additional time to complete and requires longer exposure to anesthesia (155 minutes in robotic group versus 103 minutes in laparoscopy), recovery time (21 hours in robotic group versus 50 hours in laparoscopy group) and blood loss (50 mL in robot versus 150 mL in laparoscopy) reveal a less invasive operation. Of the eight women who achieved pregnancy, five women had an uncomplicated pregnancy and delivered at 35 weeks' gestation.

Although the number of cases in our study was limited, we reported the sixth and seventh cases of achieved live pregnancy after robotic assisted abdominal cerclage in the English literature. 

## 4. Conclusion

It is now clearly established that robots have an important place in the gynecologist's armamentarium for minimal invasive surgeries; however, the long-term outcomes of several gynecologic procedures which are performed with the da Vinci surgical system have yet to be evaluated. Further randomized and controlled trial studies are warranted to determine if robotic surgery truly offers a benefit over laparoscopy in terms of surgical outcomes.
